# Volumetric imaging with homogenised excitation and static field at 9.4 T

**DOI:** 10.1007/s10334-016-0543-6

**Published:** 2016-03-19

**Authors:** Desmond H. Y. Tse, Christopher J. Wiggins, Dimo Ivanov, Daniel Brenner, Jens Hoffmann, Christian Mirkes, Gunamony Shajan, Klaus Scheffler, Kâmil Uludağ, Benedikt A. Poser

**Affiliations:** Faculty of Psychology and Neuroscience, Maastricht University, Maastricht, The Netherlands; Scannexus BV, Maastricht, The Netherlands; German Centre for Neurodegenerative Diseases (DZNE), Bonn, Germany; High Field MR Center, Max Planck Institute for Biological Cybernetics, Tuebingen, Germany; Department for Biomedical Magnetic Resonance, University of Tuebingen, Tuebingen, Germany

**Keywords:** Ultra-high field MR, Parallel transmission, B_0_ shimming, Flip-angle homogenisation, MPRAGE, 3D EPI

## Abstract

**Objectives:**

To overcome the challenges of B_0_ and RF excitation inhomogeneity at ultra-high field MRI, a workflow for volumetric B_0_ and flip-angle homogenisation was implemented on a human 9.4 T scanner.

**Materials and methods:**

Imaging was performed with a 9.4 T human MR scanner (Siemens Medical Solutions, Erlangen, Germany) using a 16-channel parallel transmission system. B_0_- and B_1_-mapping were done using a dual-echo GRE and transmit phase-encoded DREAM, respectively. B_0_ shims and a small-tip-angle-approximation kT-points pulse were calculated with an off-line routine and applied to acquire T_1_- and T_2_^*^-weighted images with MPRAGE and 3D EPI, respectively.

**Results:**

Over six in vivo acquisitions, the B_0_-distribution in a region-of-interest defined by a brain mask was reduced down to a full-width-half-maximum of 0.10 ± 0.01 ppm (39 ± 2 Hz). Utilising the kT-points pulses, the normalised RMSE of the excitation was decreased from CP-mode’s 30.5 ± 0.9 to 9.2 ± 0.7 % with all B_1_^+^ voids eliminated. The SNR inhomogeneities and contrast variations in the T_1_- and T_2_^*^-weighted volumetric images were greatly reduced which led to successful tissue segmentation of the T_1_-weighted image.

**Conclusion:**

A 15-minute B_0_- and flip-angle homogenisation workflow, including the B_0_- and B_1_-map acquisitions, was successfully implemented and enabled us to reduce intensity and contrast variations as well as echo-planar image distortions in 9.4 T images.

## Introduction

The increased signal-to-noise ratio (SNR) [1–3] and contrast-to-noise ratio (CNR) [4] of ultra-high field (UHF) MRI provide a platform for imaging at higher resolution [5], allowing both structural [6, 7], and functional [8–10] details to be observed down to cortical layers. The increased field-induced susceptibility contrast also opens up new opportunities in T_2_^*^- and susceptibility weighted imaging (SWI) as well as for quantitative susceptibility mapping (QSM) [11–14]. Novel contrast mechanisms also come into play, such as the anisotropic susceptibility of white matter [15–17].

However, in order to fully take advantage of the increased SNR and CNR in UHF MRI, especially for whole brain and body imaging, one must overcome the RF inhomogeneity at UHF. At field strengths of 3 T and beyond, the RF wavelength in vivo becomes comparable to, or smaller than, the dimension of the imaging object [18–25]. This leads to interferences in the transmitted RF (B_1_^+^) field and results in strong intensity and contrast variations in the final image [24]. The severity of the RF inhomogeneity increases as the RF wavelength reduces with field strength, e.g., from 7 T to 9.4 T [25].

These challenges can be addressed in several ways: RF coil [18], dielectric pads [26, 27], as well as RF pulse design [28] and parallel transmission with B_1_ shimming [25, 29–31] or transmit sensitivity encoding (Transmit-SENSE) [32–34] have been proposed as possible solutions to the problem of RF inhomogeneity at UHF. Small-tip-angle (STA) pulses can, for example, be designed using the spatial domain method [32] with magnitude least square optimisation [35] in combination with k-space trajectories such as kT-points [36] or spiral nonselective (SPINS) [37]. Both kT-points and SPINS pulses have been shown to be effective in ameliorating RF inhomogeneities and have been used in Magnetisation Prepared Rapid Acquisition Gradient Echo (MPRAGE) [38] for T_1_-weighted anatomical imaging [39, 40].

Benefits and challenges of UHF both include the increased effects of magnetic susceptibility and the change in relaxation times that improve various contrasts, but the downside is stronger image distortion [41], blurring and signal losses [42] due to B_0_-inhomogeneity. B_0_-inhomogeneities at UHF present a significant challenge especially in the context of echo planar imaging (EPI) [43] which is the sequence of choice in functional MRI and other rapid imaging applications. Furthermore, spatial variation in B_0_ influences the performance of the RF excitation. B_0_ variation can be alleviated with higher-order shim coils such as the 3rd and 4th orders, which have been shown to provide significant improvements in both global and local B_0_ homogeneity [44].

Here, we demonstrate a B_0_- and flip-angle-homogenisation workflow as implemented on a human 9.4 T scanner, to address the aforementioned challenges of UHF MRI. The B_0_-shimming workflow uses a field-map-based shimming technique that homogenises the static B_0_-field in a region defined by an interactively generated brain mask down to 0.12 ppm (48.0 Hz), utilising shim coils up to (partial) 3rd order. The flip-angle-homogenisation workflow takes in a B_0_ map from the shimmed field and channel-by-channel complex B_1_^+^ maps, both measured as part of the calibration routine. Parallel RF pulses are then calculated using STA approximation, which can then be applied on the scanner with modified sequences. The workflow is presented by the example of volumetric kT-points excitation pulses. Sample T_1_-weighted MPRAGE [38] and T_2_^*^-weighted 3D EPI [45] images collected in conjunction with the B_0_- and flip-angle-homogenisation routine are shown.

## Materials and methods

All experiments were performed on a 9.4 T human MR scanner (Siemens Medical Solutions, Erlangen, Germany) using a head gradient set (AC84-mk2, maximum amplitude 80 mT/m, maximum slew rate 333 T/m/s, inner diameter 36 cm) in combination with a 16-channel parallel transmission system (1 kW per channel) and a dual-row 16-channel transmit/31-channel receive array coil [46]. Online local SAR monitoring was achieved by a vendor-provided system installed on the transmit chain [47]. The SAR matrices used for the online local SAR monitoring were derived from an EM simulation [30] on an adult male model (Hugo) with safety margins (a factor of 2) and limits set according to [48] and compressed according to the virtual observation points (VOPs) method [49]. A common model was used for all subjects. The same VOPs compressed SAR matrices were used in SAR prediction in the off-line pulse calculations and simulations. All in vivo experiments were approved by the local ethics committee and performed in accordance with internal safety guidelines and all participating volunteers gave written informed consent.

### B_0_- and flip-angle-homogenisation workflow

**Fig. 1:**
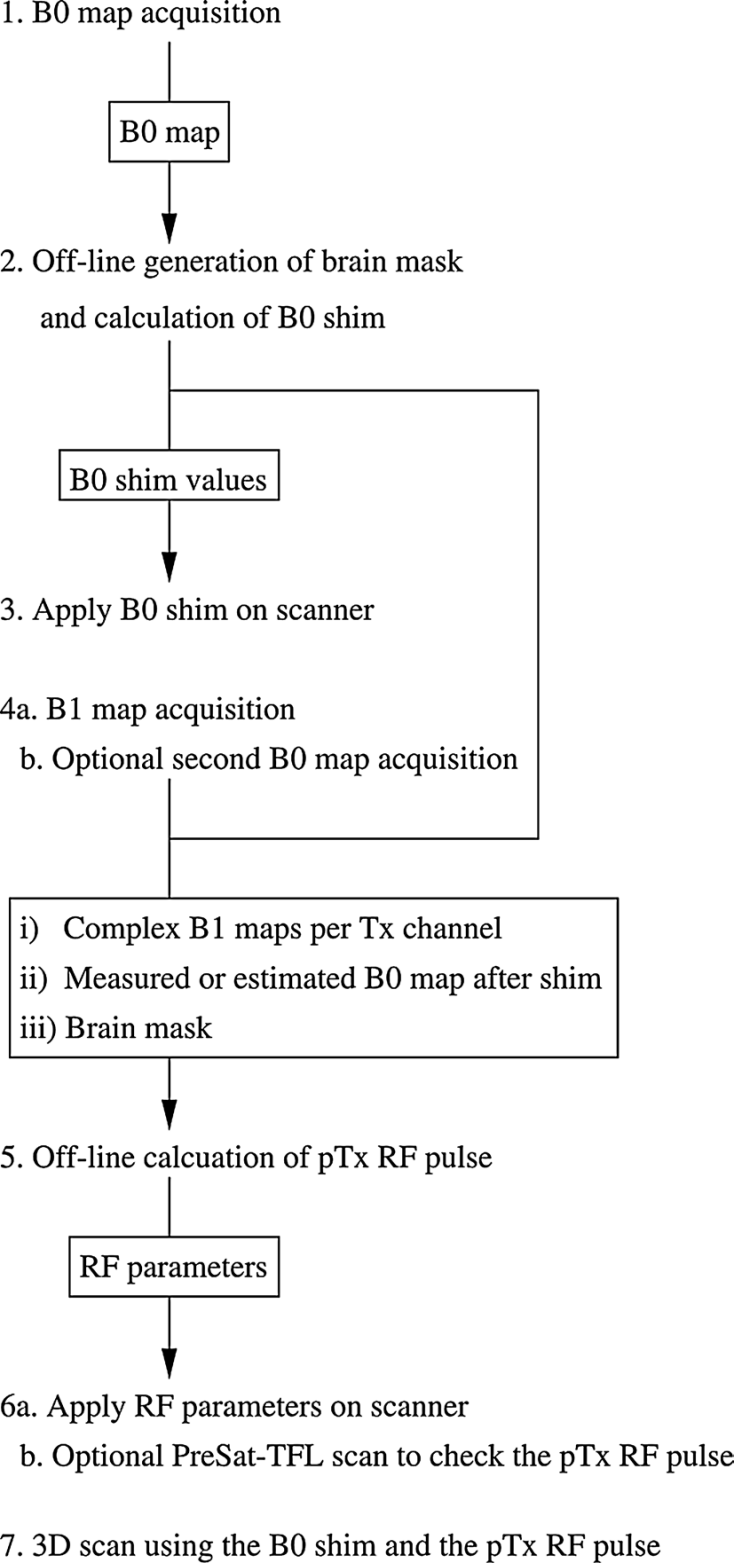
The volumetric B_0_ and flip-angle homogenisation workflow

The volumetric B_0_- and flip-angle-homogenisation workflow consists of calibration scans, B_0_-shim and RF calculations, and optional extra B_0_ and pTx RF pulse flip angle mappings for validation. The workflow is summarised in Fig. 1. All off-line calculations are carried out in MATLAB (MathWorks, Natick, MA US).

### Calibration scans

B_0_ field maps were obtained from a dual-echo 3D GRE sequence (TR = 30 ms, TE_1_ = 1.00 ms, TE_2_ = 3.21 ms, nominal flip-angle = 8 degrees, nominal voxel size = 4 mm isotropic, matrix size 50 × 50 × 44, bandwidth 1560 Hz/pixel, total scan duration 1:49 min). B_0_ shimming including some 3rd order terms (Z3, Z2X, Z2Y, ZX2Y2) was accomplished with a custom software [50]. The B_0_ shim routine unwraps the measured fieldmap [51] and fits spherical harmonics basis functions up to all 4th orders to it. Including the extra harmonics terms, it allows us to take some of the deviation of the physical shim coils from the spherical harmonics basis functions into account. The spherical harmonics terms for the shim coils had previously been determined by one-off calibration measurement, and were used to calculate the required shim currents to achieve field optimisation in the target region typically by a least-square fit to the in-session measured fieldmap. In order to take the system’s shim current limits into account, a constrained optimisation routine provided by CVX [52, 53] was used in the shim current calculation. The target region for B_0_ shimming was defined by a brain mask generated from the magnitude images of the dual-echo GRE using FSL-BET [54]. A second B_0_ field map was acquired after applying the shim values in order to take the new B_0_ field distribution into account in the RF pulse calculation. This step is optional as the B_0_ shim routine can also generate a predicted B_0_ map after shimming for later pulse calculation.

Complex B_1_ maps from all the transmit channels were obtained using a transmit phase-encoded [55], T_2_ and T_2_^*^ compensated version of DREAM [56] (imaging train repetition time = 6.8 ms, TR = 7.5 s, TE_1_ = 2.22 ms, TE_2_ = 4.44 ms, nominal imaging flip-angle = 7 degrees, nominal preparation pulse FA = 55.5 degrees, imaging slice thickness 4 mm, slice separation 10 mm, preparation pulse slice thickness 8 mm, voxel size 4 mm isotropic, matrix size 64 × 56 × 15, bandwidth 690 Hz/pixel, 32 transmit phase encode steps, total scan duration 4:00 min). After the pulse calculation (see below), the flip angle of the RF pulse was mapped by an implementation of the pre-saturation turbo-flash (PreSat-TFL) sequence [57] to verify it against the prediction of the optimisation algorithm. The PreSat-TFL method parameters were: imaging train repetition time 5.9 ms, TR 10 s, TE 2.24 ms, nominal imaging FA 8 degrees, nominal preparation pulse FA 45 degrees, imaging slice thickness 4 mm, voxel size 4 mm isotropic, matrix size = 64 × 64 × 1, bandwidth 690 Hz/pixel, total scan duration 20 s.

### RF pulse calculation

The parallel transmit excitation pulses utilised in the imaging sequences were designed using the spatial domain method [32] with magnitude least square optimisation [35] under the small tip-angle approximation. The optimisation was done using a conjugate-gradients-based algorithm [58] which includes a global SAR regularisation and a local SAR regularisation by means of a VOP-compressed SAR matrix [59]. Localised B_1_^+^ drop-out in the optimisation solution was avoided by using a region growing algorithm [60]. kT-points [36] were used as the *k*-space trajectory for the excitation pulses.

**Fig. 2:**
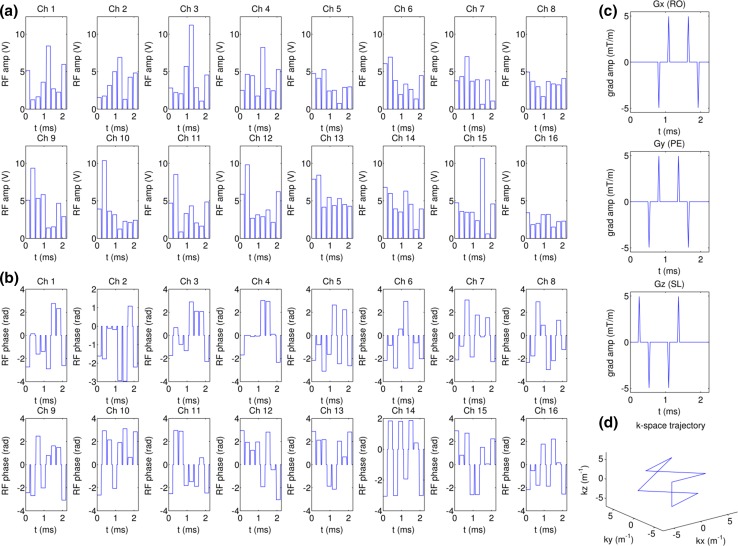
**a**, **b** Channel-by-channel RF. **a** amplitudes and **b** phases from an 8-point kT-points pulse of total duration 2.24 ms. **c** The gradient blips correspond to the 8-point kT-point trajectory. **d** The *k*-space trajectory of the 8-point kT-points pulse which starts and ends at the *k*-space centre

The kT-points trajectory consists of six equidistant points placed on the *kx*, *ky* and *kz* axes at ±6.33 m^−1^, which is roughly the inverse of the RF wavelength at 9.4 T [61], plus two additional points at the *k*-space centre at the beginning and the end of the trajectory. This trajectory equates to eight rectangular sub-pulses of duration 210 us interleaved with 70 us gradient blips. The RF waveform was divided into rectangular sub-pulses and played out between the gradient blips. The overall pulse duration was 2.24 ms. The trajectory, gradient blips and a RF pulse are shown in Fig. 2.

### Imaging scans

MPRAGE scans were acquired using: TR = 3.75 s, TE = 3.64 ms, TI = 1.2 s, matrix size 384 × 384 × 256, voxel size 0.6 mm isotropic, sagittal slices and anterior-to-posterior phase encode direction, slice partial Fourier 7/8, echo train length = 2.1 s, nominal flip-angle = 5 degrees, GRAPPA parallel imaging using factor 3 along the in-plane phase encoding direction with 24 reference lines, bandwidth 180 Hz/pixel; total scan duration was 8:58 min. The T_1_-weighting in MPRAGE was achieved by an adiabatic inversion pulse. Here, the same complex RF waveform was applied in all transmit channels, i.e., only the channel relative phases were adjusted to achieve a CP-like mode of the coil. The nominal flip-angle of the CP-mode pulse was defined as the average flip angle in a central region around the bright CP-mode spot in the middle of the brain. The inversion pulse used in the experiment was a 13 ms TR-FOCI adiabatic pulse with a peak voltage of 79.8 V applied to each transmit channel (yielding a nominal peak B_1_ amplitude of 10 uT) [62]. An additional proton-density-weighted image was acquired for receive bias-field correction by switching off the inversion pulse and minimising TE and TR while keeping the other parameters unchanged. For one-off comparison, the same acquisitions were repeated using a standard non-selective RF excitation pulse of 0.10 ms duration in the same CP-like mode as used for the TR-FOCI inversion pulse. For this set of MPRAGE sequence parameters, the maximum 10 g averaged local SAR per TR estimated by using the VOPs compressed SAR matrices were 0.77 and 1.11 W/kg for the kT-points and the CP-like mode excitation pulses, respectively, and was 6.77 W/kg for the TR-FOCI inversion pulse.

The T_1_-weighted MPRAGE image was segmented into grey matter, white matter and cerebrospinal fluid maps with FSL-FAST [63] in a sequence of customised steps. First, the T_1_-weighted image was realigned rigidly to and divided by the proton-density image to remove any intensity bias introduced by receive field inhomogeneity [64]. Second, the co-registered proton-density image was corrected for bias fields with FSL-FAST and then passed to FSL-BET to create a brain mask. Third, the brain mask was then used to extract the brain region from the bias-corrected T_1_-weighted image. Finally, the brain extracted T_1_-weighted image was passed to FSL-FAST for tissue segmentation.

The same kT-points excitation was also applied in a 3D EPI sequence [45] with parameters as follows: TR = 61 ms, effective volume TR = 12.7 s, TE = 22 ms, matrix size 256 × 256 × 208, voxel size 0.75 mm isotropic, phase partial Fourier 6/8, nominal flip-angle 15 degrees, bandwidth 1396 Hz/pixel, and parallel imaging undersampling with GRAPPA factor 3 along the in-plane phase-encoding direction using 96/48 reference lines/partitions that were acquired in a segmented lines-in-partition order [65]. The total acquisition time was 1:28 min. This acquisition was also repeated in a CP-like mode transmission using a 1.0 ms duration rectangular pulse. With the given 3D EPI sequence parameters, the maximum 10 g averaged local SAR per TR were estimated to be 1.56 and 1.12 W/kg for the kT-points and the CP-like mode excitation pulses, respectively. The 3D EPI sequence was implemented such that each excitation yielded a *kx*-*ky* plane at a different *kz* increment, using linear ordering. Each readout was preceded by a three-lobed non-phase-encoded navigator readout at minimum TE, which served the dual purpose of EPI eddy current (Nyquist N/2 ghost) correction [66] and correction of B_0_-drift due to subject breathing during the acquisition [67].

## Results

**Fig. 3:**
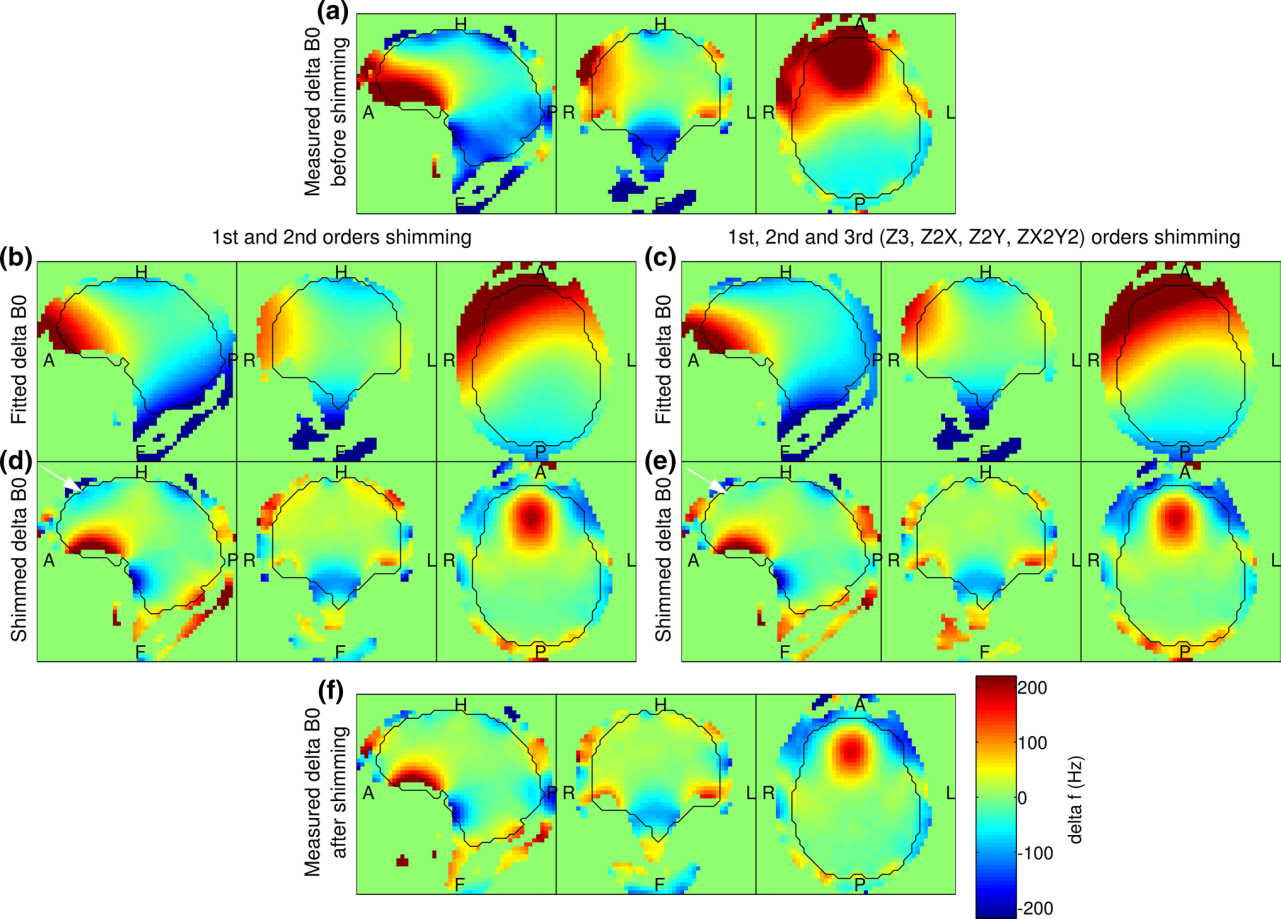
**a** B_0_ map derived from a 3D dual-echo GRE under the tune-up shim setting. **b** The fitted B_0_ using the available fields from all the 1st and 2nd order shim coils. **c** The fitted B_0_ field using all the available fields from the 1st and 2nd order shim coils, and four 3rd order shim coils: Z3, Z2X, Z2Y, ZX2Y2. **d** Prediction of the shimmed B_0_ field using shim coils up to 2nd order. **e** Prediction of the shimmed B_0_ field using all available shim coils up to the 3rd order. The *white arrows* in the sagittal view of **d**, **e** indicate the difference in the shim results in the frontal lobe. **f** B_0_ map measured after applying the shim values from **e**

Figure 3 shows the B_0_ shimming results from an example set of in vivo measurement using the shim routine described above. Using only 1st and 2nd order shim coils, shimming reduces the standard deviation of the B_0_ field within the brain mask to 50.1 Hz with a 90th percentile range of 148.0 Hz; when adding the four available 3rd shim coils (Z3, Z2X, Z2Y, ZX2Y2), the standard deviation and the 90th percentile range were reduced to 46.1 and 138.6 Hz, respectively.

**Fig. 4:**
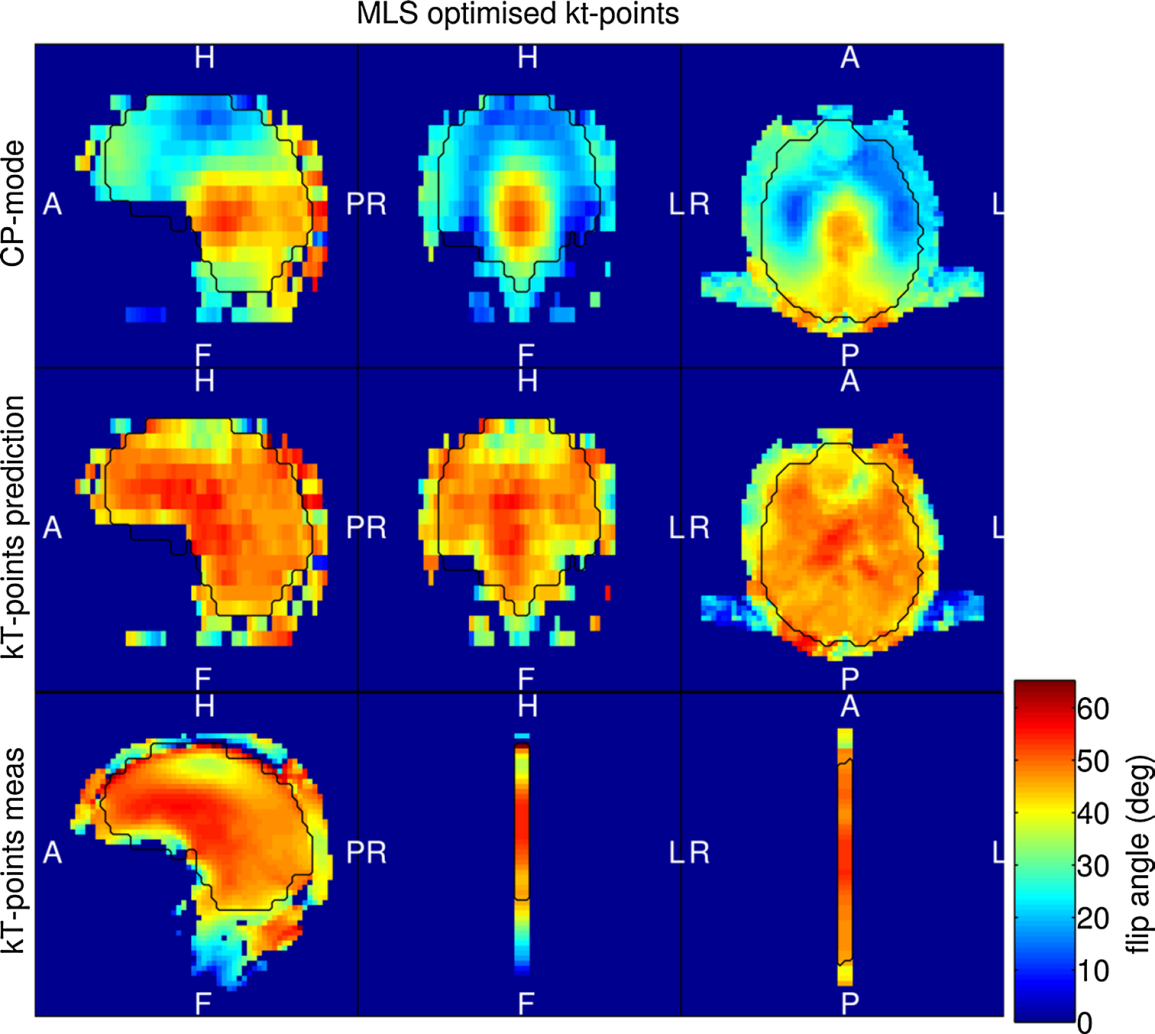
*Top row* CP-mode B_1_^+^ magnitude distribution; *middle row* MLS optimised 8-point kT-points pulse flip-angle distribution; *bottom row* the flip angle distribution of the kT-points pulse measured by a one-slice PreSat-TFL in sagittal orientation. The target flip-angle set in the PreSat-TFL protocol was 45 degrees

Illustrations of the flip-angle homogenisation from the same measurement session are shown in Fig. 4. The top row shows the B_1_^+^ magnitude distribution of the CP-like-mode, which has a normalised RMSE of 33.3 %. The middle row of Fig. 4 is the flip-angle prediction of the MLS optimised 8-point kT-points pulse, which has a normalised RMSE of 9.1 %, and the bottom row is the pulse’s corresponding single-slice flip-angle distribution measured by the PreSat-TFL in sagittal orientation. In both B_0_ and flip-angle homogenisation, the measured B_0_ and the flip angle distribution visually agrees very well with their predictions.

**Fig. 5:**
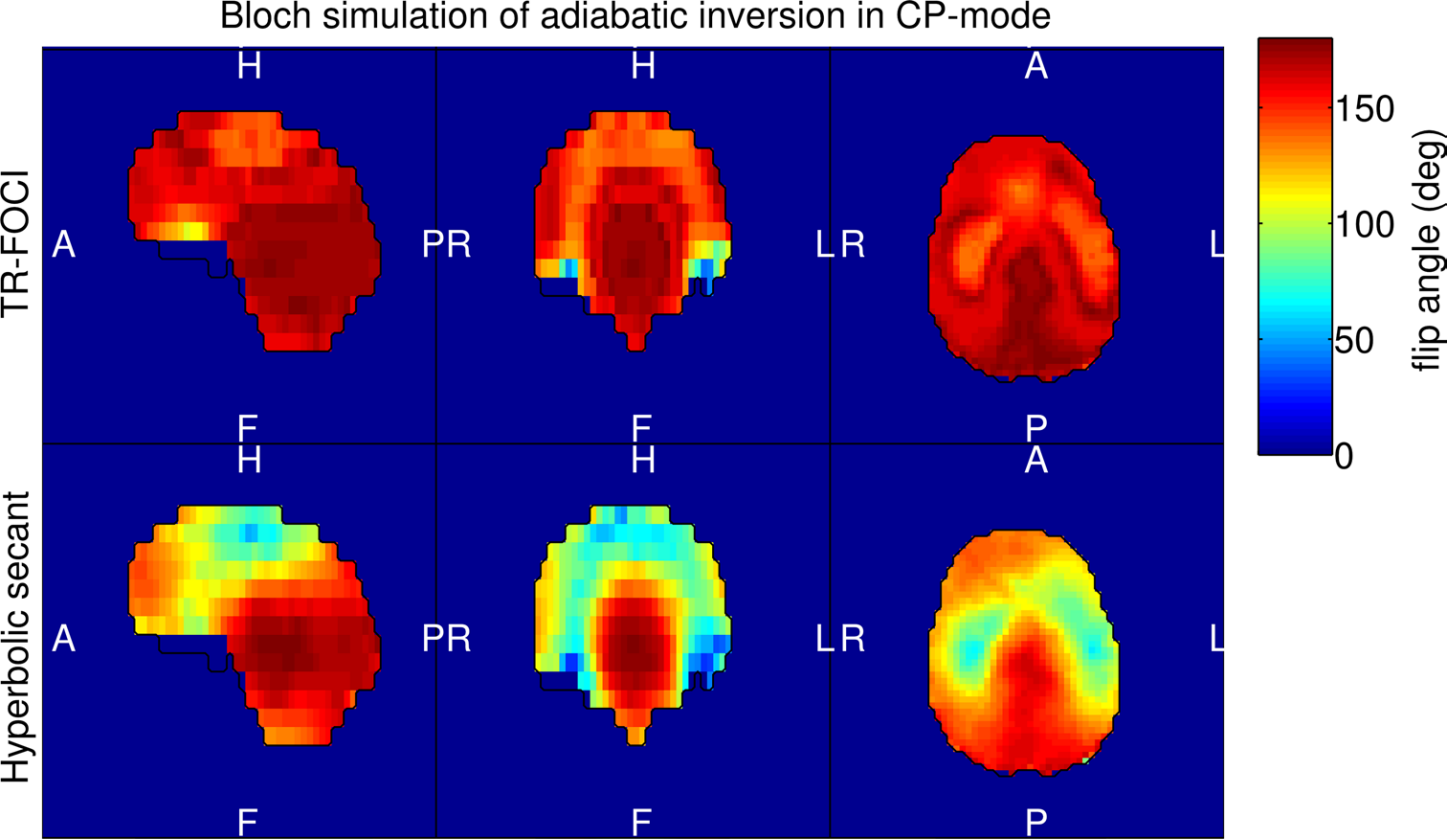
Achieved flip-angle as result of Bloch simulation for *top row* TR-FOCI adiabatic inversion pulse (79.8 V); *bottom row* standard hyperbolic secant adiabatic inversion pulse (166.0 V)

The top row of Fig. 5 shows the post hoc Bloch simulations of the TR-FOCI adiabatic pulse and the vendor’s standard 5.12 ms duration hyperbolic-secant adiabatic pulse [68]. They were simulated using the subject-specific B_0_ and B_1_ maps collected in the same session. The pulse amplitude of the TR-FOCI and the hyperbolic secant inversion pulses were 79.8 and 166.0 V, respectively. The lower adiabatic threshold of the TR-FOCI allowed it to achieve at least partial magnetisation inversion in regions with low field intensities in the CP-mode pattern. In contrast, large regions of brain, especially near the temporal lobes, were not inverted by the hyperbolic secant adiabatic pulse. The normalised RMSE of the TR-FOCI and hyperbolic secant inversions were 10.6 and 26.5 %, respectively. Given this superior inversion performance of the TR-FOCI pulse it was used in all the following MPRAGE measurements.

**Fig. 6:**
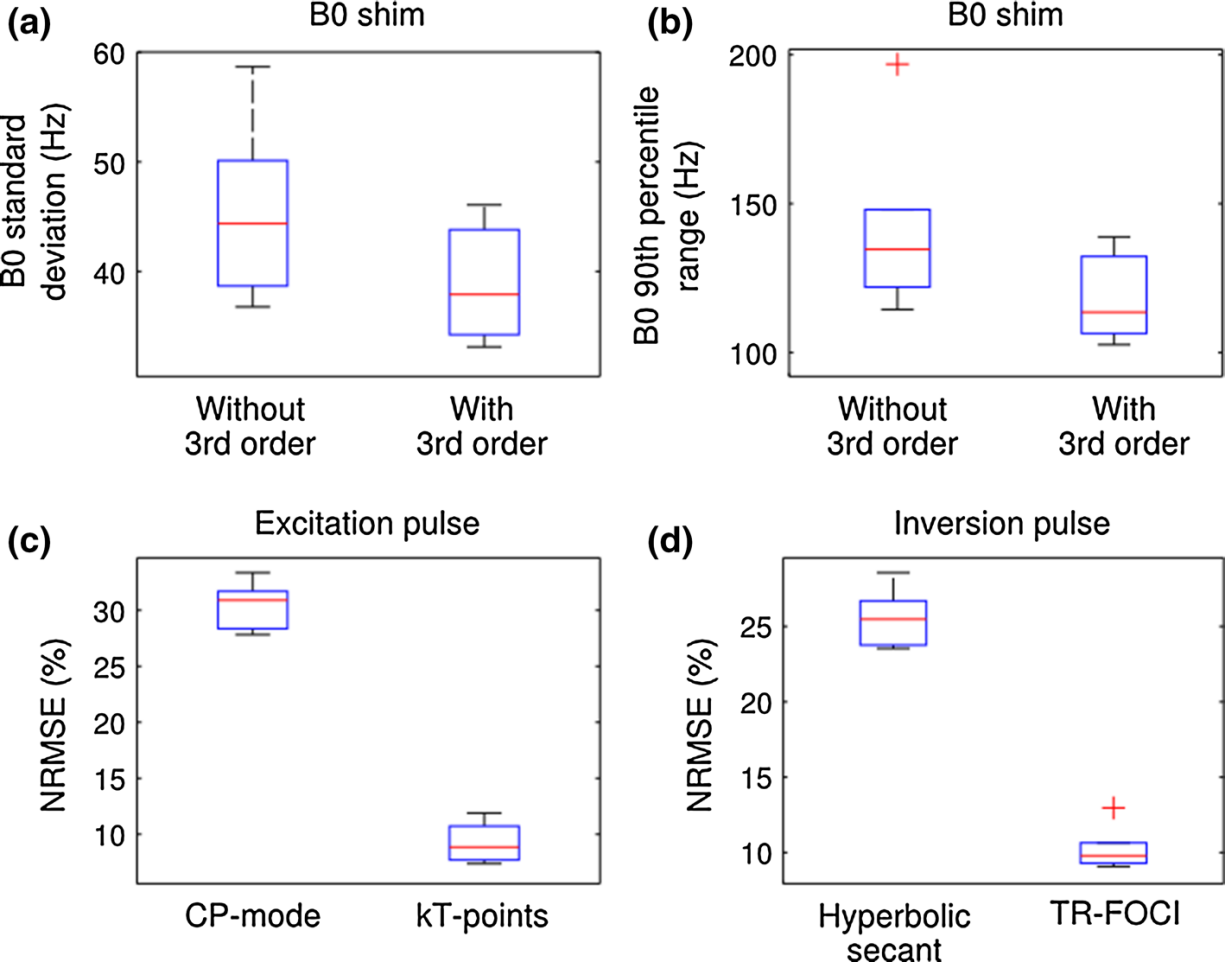
*Box plots* of **a** the standard deviation and **b** the 90th percentile range of B_0_ distributions after shimming with and without including the 3rd order coils; and the normalised RMSE of **c** the CP-mode and kT-points excitation pulses and **d** the Hyperbolic secant and TR-FOCI inversion pulses. In *each box*, the central mark is the median; the *edges of the box* are the 25th and 75th percentiles. The *whiskers* extend to the most extreme values which are not outliers, and the *outliers* are plotted as *red crosses* individually

The B_0_ shim and RF pulses performances over a set of 6 in vivo measurements are summarised Fig. 6. On average, using only 1st and 2nd orders shim coils, the standard deviation and the 90th percentile range of the B_0_ distribution after shim were 45.5 ± 3.2 and 141.7 ± 12.0 Hz, respectively. Including the 3rd order coils reduced them to 38.8 ± 2.1 and 117.9 ± 6.1 Hz, respectively. The average normalised RMSE of the excitation pulses were 30.5 ± 0.9 % and 9.2 ± 0.7 %, respectively, for CP-mode and kT-points; and they were 25.6 ± 0.9 and 10.3 ± 0.6 %, respectively, for hyperbolic secant and TR-FOCI inversion pulses.

**Fig. 7:**
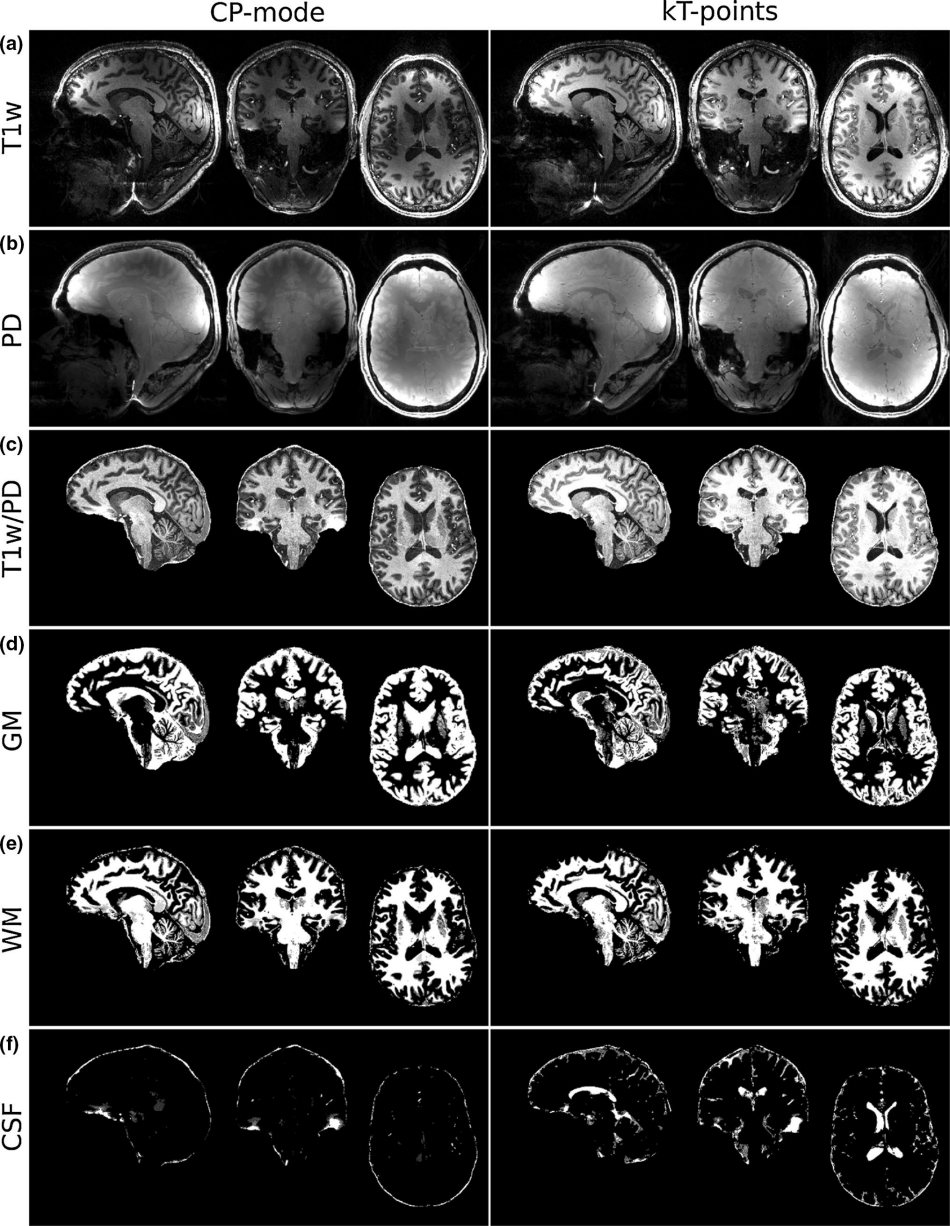
**a** T_1_-weighted MPRAGE image using TR-FOCI inversion and CP-mode excitation (*left column*) and kT-points excitation (*right column*). The intensities in both columns are scaled identically. **b** Proton density image using kT-points. **c** Brain extracted and receive bias corrected T_1_-weighted image from **a** and **b**. **d**–**f** Grey matter, white matter and cerebrospinal fluid tissue probability maps extracted from **c** using FSL-FAST

Figure 7a and b show the T_1_-weighted MPRAGE images using TR-FOCI inversion with CP-mode (left column) and kT-points (right column) excitation pulses for the imaging trains. The T_1_-weighted and the proton density images were co-registered and divided to remove the receive bias fields. The brain-extracted and receive-bias-free images are displayed in Fig. 7c). These images were segmented by FSL-FAST into grey matter, white matter and cerebrospinal fluid maps which are shown in Fig. 7d, e and f, respectively.

B_1_^+^ voids, SNR and contrast variations due to B_1_^+^ inhomogeneity can be seen in the CP-mode T_1_-weighted and proton density images. Consequently, in several regions proper segmentation of grey matter and CSF was not possible.

**Fig. 8:**
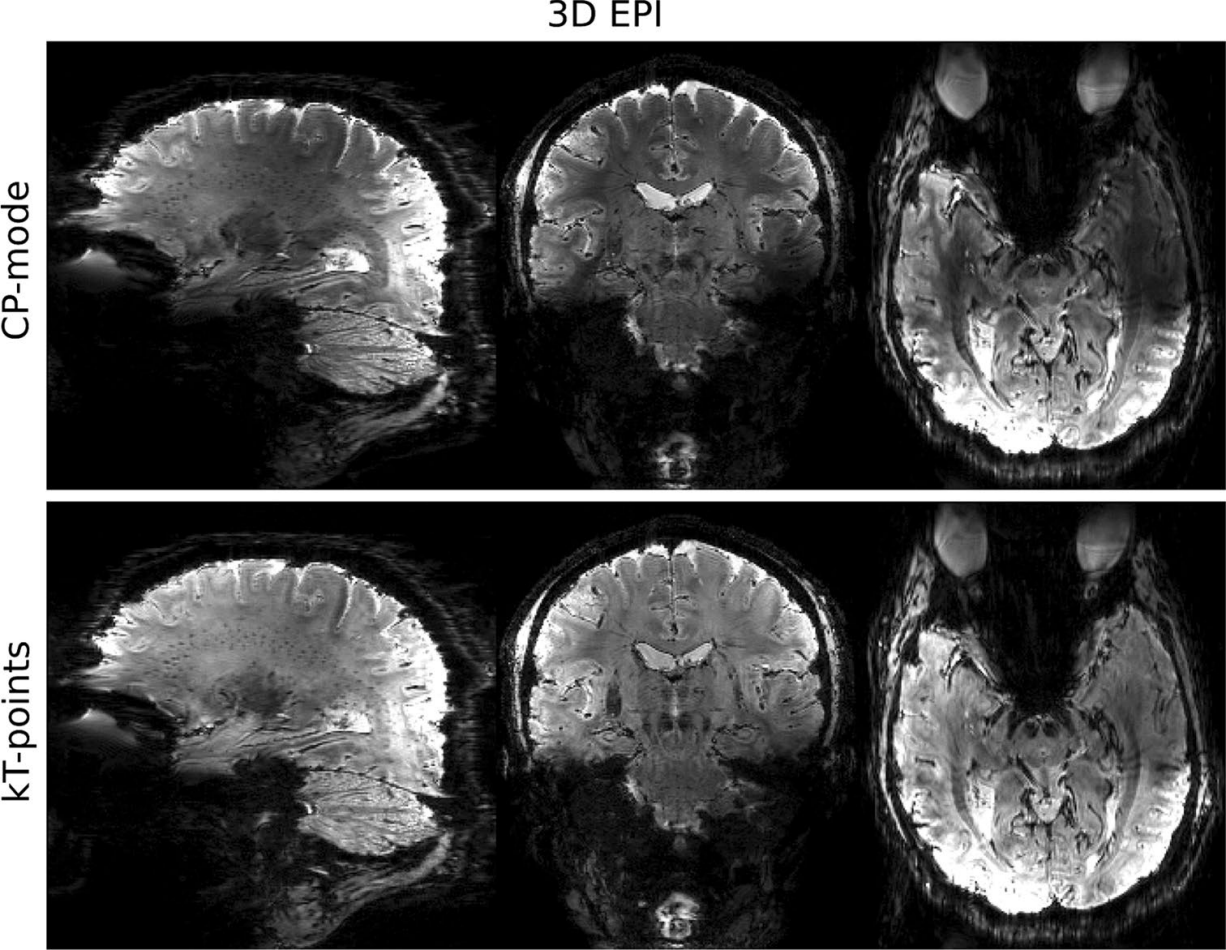
3D EPI images at 0.75 mm isotropic resolution, obtained using CP-mode (*top row*) and kT-points (*bottom row*) excitations

Results of the 3D EPI scan are shown in Fig. 8. The top and bottom rows are 3D EPI images obtained using CP-mode and kT-points excitations, respectively. Similar to the MPRAGE images, signal and contrast variations can be seen in the CP-mode image.

## Discussion

B_0_- and B_1_-inhomogeneities are the well-known challenges in UHF MRI. Here, we presented the workflow used at our institution to homogenise both B_0_ and the excitation flip-angle, allowing us to acquire high quality volumetric images at 9.4 T and further explore the potential of UHF imaging. The entire B_0_ and B_1_ calibration workflow currently takes approximately 15 min in total, including the (~6 min) B_0_ and B_1_ maps acquisitions. With further developments such as incorporating the B_0_ and B_1_ mapping into the scanner’s on-line image reconstructions and further streamlining the data transfer between the scanner’s host computer and the PC on which the B_0_ and B_1_ shim calculations are performed, we expect that the total calibration time can be shortened to approximately 8 min. This is only a small proportion of time typically allocated for UHF MRI studies.

The first benefit that the homogenisation workflow can bring to single- and multi-channel transmission systems alike is the potential reduction in signal loss, T_2_^*^ blurring and distortion due to B_0_ inhomogeneity, especially for the case of EPI [69]. By using a ‘brain-only’ region of interest and precisely calibrated shim coils, the B_0_ linewidth in the brain was brought down to 39 ± 2 Hz (0.10 ± 0.01 ppm). Using the four additional 3rd order shim coils, the linewidth and the 90th percentile of the B_0_ field distribution in the brain were on average further reduced by 14.7 and 16.9 %, respectively. This improvement is most apparent in the frontal regions of the brain as shown in Fig. 3d, e.

The second benefit of the homogenisation workflow is the reduction in signal and contrast variations across the image due to B_1_^+^ inhomogeneity which is a bigger challenge than B_0_ homogenisation at 9.4 T. A previous simulation study on the same coil at 9.4 T has shown the limited capability of flip-angle homogenisation using static shim only [30]. By utilising the full 16-channel parallel RF transmission system dynamically, we were able to design kT-points pulse in the same brain region of interest to reduce the normalised RMSE of the excitation flip angle on average by 69.7 %, in comparison to CP-mode. All B_1_^+^ voids could be eliminated, as can be observed in Fig. 4. The PreSat-TFL validation scans confirmed the agreement between the predicted and achieved flip angle distribution, and implicitly also the correct delivery of the pulse (e.g., gradient axis orientation, channel ordering, and phase sign).

The results over 6 scan sessions demonstrated that the improvements on both the B_0_ and flip-angle homogenieties are consistent and reproducible. Various parts of the B_0_ and flip-angle homogenisation procedure work in synergy with each other. Apart from reducing artefacts, a homogenised and known B_0_ distribution can also ease the RF optimisation by narrowing the range of off-resonance in the problem. By excluding the subcutaneous tissue and the neck with the brain mask, the size of the RF optimisation problem is reduced and hence improves its speed and memory usage.

RF exposure to volunteers is a major concern in UHF MR, especially in combination with the use of parallel transmission. Using EM-simulations generated SAR matrices and VOPs compression, local SAR can be regularised during the pulse design process [59]. With the knowledge of the desired sequence parameters, the maximum local SAR can also be predicted in the pulse design workflow for a particular run of sequence [70]. The off-line maximum local SAR look-ahead allows the scanner operators to adjust the sequence parameters in order to keep the 10 s and 6 min exposure below the 20 W/kg and the 10 W/kg limits, respectively, as described in IEC 60601-2-33.

Several methods for B_0_ and B_1_ mapping have been shown in the literature for B_0_ shim and RF pulse optimisations. It is out of the scope of this article to review or evaluate them, and the choices here were made based on pragmatic criteria: ease of implementation and effectiveness for the experimental setting (whole brain B_1_ and B_0_ shim). Shim current calculation for B_0_ shims can, in principle, be achieved with a least-square fit expressed as a matrix inversion. However, the physical shim current limits need to be taken into account, for which we have chosen a constrained optimisation approach instead. For B_0_ mapping, we have found that a standard dual-echo 3D GRE sequence, with phase unwrapping as shown in Ref [51], was fully sufficient for application in the brain, without apparent artefacts. For the RF optimisation, the main goal was in homogenising a low flip angle excitation pulse and we have hence chosen the STA approximation approach with a magnitude least square cost, due to its ease of obtaining a magnitude homogenised solution with a potential reduction in global RF power [35].

Two B_1_ mapping sequences were use in this study, DREAM and PreSat-TFL. They were chosen for a couple of different aspects of B_1_ mapping based on their different desired properties. The individual transmit channel B_1_ maps were acquired with transmit phase encoded DREAM because of its speed, its insensitivity to B_0_ variation and more robust against motion in comparison to PreSat-TFL. However, due to the RF pulse timing and layout in DREAM, it is not straightforward to map the kT-points pulses with it. Hence, a 2D PreSat-TFL, which is more flexible in terms of the mapping RF pulse, was used as the optional fast (20 s) qualitative validation of the designed pTx pulse. A full 3D quantitative mapping of the kT-points pulse is also possible by using an AFI [71] sequence, but this is not included in the current workflow because of its long acquisition time (~3 min).

The B_0_ and B_1_ maps collected during the session were also used in a Bloch simulation to decide which type of adiabatic pulse and what voltage amplitude was suitable for inversion in our imaging experiments. Our simulations have shown that the TR-FOCI inversion pulse performed better than the hyperbolic secant pulse provided in the standard product sequence, despite only using half the maximum transmitter voltage. Hence, TR-FOCI was chosen as the inversion pulse in our T_1_-weighted MPRAGE for structural imaging and tissue segmentation. Despite its low adiabatic threshold, the TR-FOCI pulse in combination with a CP-like mode in this particular setup still could not provide a complete inversion throughout the region of interest defined by the brain mask. Using a large tip angle design as reported by [39, 72], the inversion efficiency can potentially be further improved. The feasibility of incorporating this approach into our B_0_- and flip-angle-homogenisation is being investigated.

The efficient inversion of TR-FOCI in combination with the homogenised excitation through a kT-points pulse have led to significant improvement in the quality of the T_1_-weighted MRRAGE images acquired at 9.4 T. As can be seen in the comparison of CP-mode and kT-points acquisitions of the T_1_- and proton density weighted images shown in Fig. 7, the variations in intensity and contrast across the image were largely eliminated by substituting the standard rectangular pulses by parallel transmit kT-points excitations. Successful segmentation of the receive-bias-corrected images was achieved without further adjustment or manual intervention; this was not the case for the CP-mode acquired image.

Similar improvements were observed for the 3D EPI when comparing the CP-mode and kT-points acquired images. Similar improvements were observed for the 3D EPI when comparing the CP-mode and kT-points acquired images. While a 0.1 ms rectangular pulse is typically used in an MPRAGE readout train, a 1 ms pulse was used for the 3D EPI CP mode acquisition, because of the higher required flip angle and the lack of a time constraint. This “stretching” of the pulse proportionately reduced the required pulse amplitude, which explains the inversion of the local SAR estimates between the CP-mode and kT-points protocols for MPRAGE and 3D EPI listed in the Materials and Methods section. The increased pulse duration reduces the pulse bandwidth, which however remained much higher than the B_0_ variations within the brain mask. Therefore most of the excitation inhomogeneity can be attributed to B_1_ inhomogeneity from the transmit coils at this particular field strength.

Application of 3D EPI at 3 T and above has recently been receiving increased attention not only for BOLD imaging [45, 73, 74] but has also been shown to be an attractive choice for rapid acquisition of T_2_^*^-weighted structural data [75]. This can readily be extended to the application of SWI and QSM, as has recently been demonstrated at 3 T [76], 7 T and 9.4 T [77]. The benefits of 3D EPI are greatest at high-resolution which is particularly beneficial at UHF. A tremendous advantage of 3D sequences with two phase encoding directions is that they lend themselves to efficient parallel imaging undersampling strategies such as CAIPIRINHA [78] as has been shown also in the context of 3D EPI [79, 80].

The B_0_ shim and RF pulse calculated by the B_0_- and flip-angle-homogenisation workflow can be applied to other 3D scans such as GRE. The volumetric workflow has been extended to design slice-specific spokes pulses for 2D excitations as demonstrated in [81] for high in-plane resolution GRE imaging.

## Conclusion

In summary, we demonstrated a B_0_- and flip-angle-homogenisation procedure that currently takes approximately 15 min to run, and homogenises B_0_ linewidth down to 0.10 ± 0.01 ppm and flip-angle normalised RMSE down to 9.2 ± 0.7 % in the same whole brain region of interest. This enables us to reduce intensity and contrast variations as well as distortions in our 9.4 T images.
